# Yeast Infections after Esophagectomy: A Retrospective Analysis

**DOI:** 10.1038/s41598-020-61113-z

**Published:** 2020-03-09

**Authors:** Marjolein Heuker, Usma Koser, Alewijn Ott, Arend Karrenbeld, Jan Maarten van Dijl, Gooitzen M. van Dam, Anne Marie G. A. de Smet, Marleen van Oosten

**Affiliations:** 1 Department of Medical Microbiology, University of Groningen, University Medical Center Groningen, Hanzeplein 1, PO Box 30001, 9700 RB Groningen, The Netherlands; 2 Department of Critical Care, University of Groningen, University Medical Center Groningen, Hanzeplein 1, PO Box 30001, 9700 RB Groningen, The Netherlands; 3 Department of Medical Microbiology, Certe, PO Box 909, 9700 AX Groningen, The Netherlands; 4 Department of Pathology, University of Groningen, University Medical Center Groningen, Hanzeplein 1, PO Box 30001, 9700 RB Groningen, The Netherlands; 5 Department of Surgery, Division of Surgical Oncology, University of Groningen, University Medical Center Groningen, Hanzeplein 1, PO Box 30001, 9700 RB Groningen, The Netherlands

**Keywords:** Fungal infection, Risk factors

## Abstract

Esophageal malignancy is a disease with poor prognosis. Curative therapy incorporates surgery and is burdensome with high rates of infection morbidity and mortality. The role of yeast as causative organisms of post-esophagectomy infections is poorly defined. Consequently, the benefits of specific antifungal prophylactic therapy in improving patient outcome are unclear. Therefore, this study aimed at investigating the incidence of yeast infections at the University Medical Center Groningen among 565 post-esophagectomy patients between 1991 and 2017. The results show that 7.3% of the patients developed a yeast infection after esophageal resection with significantly increased incidence among patients suffering from diabetes mellitus. For patients with yeast infections, higher Acute Physiology and Chronic Health Evaluation (APACHE) II scores, more frequent intensive care unit readmissions, prolonged hospital stays and higher mortality rates were observed. One-year survival was significantly lower for patients with a yeast infection, as well as diabetes mellitus and yeast-positive pleural effusion. We conclude that the incidence of yeast infections following esophagectomy is considerable, and that patients with diabetes mellitus are at increased risk. Furthermore, yeast infections are associated with higher complication rates and mortality. These observations encourage further prospective investigations on the possible benefits of antifungal prophylactic therapy for esophagectomy patients.

## Introduction

Esophageal cancer is an aggressive disease associated with poor prognosis and a 5-year survival of 10%^1,2^. The curative treatment, when applicable, is neo-adjuvant chemoradiotherapy with surgical resection and radical lymphadenectomy. Although some benign disorders can result in esophagectomy, esophageal malignancy is the main reason for resection. Despite medical, surgical, nutritional and critical care advances, esophagectomy remains an operative intervention with high morbidity and mortality even when compared to other complex surgical procedures. Mortality rates vary depending on many factors and have been reported as 5–10%^3–5^. However, morbidity occurs in over 50% of cases and pulmonary complications, are almost universally seen as the greatest post-operative challenge for healthcare providers^6^. Moreover, patients who suffer from pneumonia show a seven-fold increase in mortality^7^. Causative microorganisms for pulmonary infection usually belong to the oropharyngeal flora, as this is likely the result of cervical anastomosis leakage.

Risk factors for yeast infections can be split into host, pathogen and health-care associated aspects. In the former category, immune-compromising conditions, older age, malignancy, corticosteroid or cytotoxic therapy and neutropenia have all shown to increase the risk of invasive *Candida* infections^8^. Mechanically ventilated, critically ill patients suffer from colonization with *Candida* species in 25% of cases, whereas colonization occurs in 50% of the patients suspected of ventilator-associated pneumonia^9^. Nevertheless, pneumonia caused by *Candida* is rare^10^. On the other hand, systemic yeast infections are associated with increased morbidity and mortality^9,11^. Complex diagnostics and clinical uncertainty in these, often critically ill patients, can lead to at least a challenging management course and at worst futile demise. Therefore, a prophylactic treatment strategy for high-risk patients may be of benefit and result in improved clinical progress and overall patient outcomes^11^.

Several studies have suggested selective decontamination of the digestive tract (SDD) and selective oropharyngeal decontamination (SOD) can reduce the (pulmonary) infection rates and improve survival in surgical and non-surgical intensive care unit (ICU) patients^12–15^. The selective decontamination principle is based on eradication of certain pathogens (i.e. aerobic Gram-negative bacteria, *Staphylococcus aureus* and yeast), resulting in fewer ICU-acquired infections. In 2011, a double-blind randomized controlled trial was carried out to evaluate the effect of SDD (a regimen consisting of oral, non-absorbable paste and enteral antibiotics including amphotericin B, tobramycin, colistin and intravenous cefotaxime or other third generation cephalosporins) or placebo in addition to standard antibiotic prophylaxis in patients receiving elective gastrointestinal surgery^16^. SDD in addition to the standard intravenous antibiotics reduced the incidence of postoperative infections and anastomotic leakage.

Given the serious implications of systemic yeast infections, the effects of SDD should be investigated in more depth in esophagectomy patients to identify whether this patient group may also benefit from tailored SDD^15–17^. However, thus far it is not known to what extent esophagectomy patients develop yeast infections and whether addition of an antifungal to a topical antibacterial application of polymyxin B and tobramycin pre-operatively may be useful as an infection prevention measure. Therefore, in this study, we retrospectively analyzed yeast infections, clinical and demographic data and complications in patients following esophageal resection at the University Medical Center Groningen (UMCG).

## Results

### Patient characteristics and outcomes

**Figure 1:**
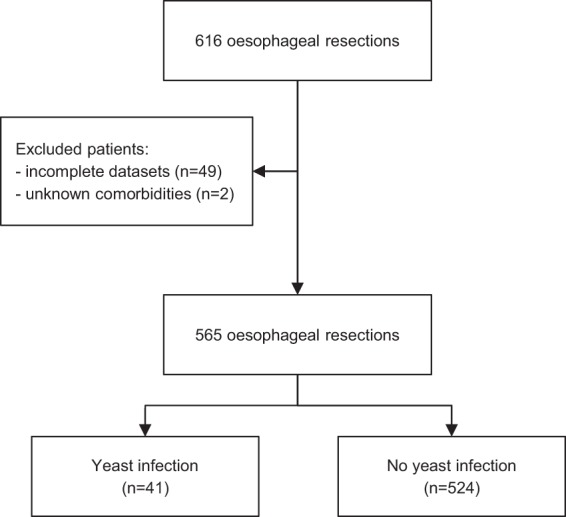
Flowchart of the study design.

The investigated patient cohort consisted of 565 patients who underwent esophageal resection in the UMCG between January 1991 and July 2017 (Fig. 1). More males underwent esophagectomy (sex ratio M:F, 4:1) and the mean age of this study population was 63.8 ± 9.1 years (mean ± SD). Resection was performed via minimal invasive esophagectomy (MIE) in 8.3% (n = 47), via an open procedure in 90.1% (n = 509), and by colon interposition (n = 9) in 1.6% of the cases (Table 1). 7.3% (n = 41) of the patients developed a yeast infection after surgery. In one patient the diagnosis of yeast infection was made at autopsy, and this was in accordance with the clinical features. Patients with diabetes mellitus (DM) developed a yeast infection significantly more often than patients without (odds ratio (OR) 3.4; 95% confidence interval (CI): 1.6–7.0; p = 0.001; Table 1). A higher APACHE II score (p = 0.028), readmission to the ICU (OR 6.7; 95% CI: 3.5–13.0; p < 0.001) and longer hospitalization (p < 0.001) were all associated with yeast infection (Table 1). Other comorbidities and characteristics of the group with yeast infection and the group with no yeast infection are displayed in Table 1.

**Table 1:** Patient characteristics.

Variable	Yeast infection	No yeast infection	P value
*Demographic characteristics*	n = 41	n = 524	
Age during surgery	64.8 ( ± 8.9)	63.8 ( ± 9.1)	0.497
Gender			0.155
Male	36 (87.8%)	411 (78.3%)	
Female	5 (12.2%)	113 (21.7%)	
*Comorbidities*	n = 41	n = 524	
HIV infection	0 (0.0%)	0 (0.0%)	
Chronic renal insufficiency	0 (0.0%)	7 (1.3%)	1.000
Chronic respiratory insufficiency	0 (0.0%)	2 (0.4%)	1.000
Cirrhosis	1 (2.4%)	6 (1.1%)	0.412
COPD	7 (17.1%)	53 (10.1%)	0.184
CVA	3 (7.3%)	18 (3.4%)	0.190
CVD	1 (2.4%)	38 (7.3%)	0.501
DM	12 (29.3%)	57 (10.9%)	0.001*
Immunological insufficiency	9 (22.0%)	134 (25.6%)	0.608
*Diagnosis*	n = 41	n = 524	
Esophageal cancer	38 (92.7%)	506 (96.6%)	0.190
Other malignancy	2 (4.9%)	16 (3.05%)	0.381
No malignancy	1 (2.4%)	2 (0.4%)	0.203
*Type of esophageal carcinoma*	n = 38	n = 506	
Adenocarcinoma	30 (79.0%)	411 (81.2%)	0.711
Squamous cell carcinoma	8 (21.1%)	82 (16.2%)	0.438
Other carcinoma	0 (0.0%)	3 (0.6%)	1.000
Dysplasia	0 (0.0%)	10 (2.0%)	1.000
*Patients with no esophageal cancer were excluded (n* = *21, with yeast infection n* = *3 and with no yeast infection n* = *18)*.			
*Type of surgery*	n = 41	n = 524	
Open esophagectomy	39 (95.1%)	470 (89.7%)	0.413
MIE	1 (2.4%)	46 (8.8%)	0.238
Colon interposition	1 (2.4%)	8 (1.5%)	0.495
*Mortality*	n = 41	n = 522	
30D	5 (12.2%)	21 (4.0%)	0.039*
3 M	9 (22.0%)	33 (6.3%)	0.002*
1Y	21 (51.2%)	118 (22.6%)	<0.001*
*Patients with unknown date of death were excluded (n* = *2)*.			
*Yeast colonization*	n = 30	n = 281	
	16 (53.3%)	106 (37.7%)	0.096
*Patients without cultures were excluded (n* = *254)*.			
*APACHE scores*	n = 24	n = 258	
II	13.5 ( ± 4.1)	12.6 (±3.7)	0.028*
IV	n = 26	n = 269	
	50.4 ( ± 17.8)	42.2 (±13.9)	0.348
*Patients with unknown APACHE II (n = 281) and APACHE IV (n* = *270) scores were excluded*.			
*Readmission to ICU*	n = 41	n = 524	
	22 (53.7%)	77 (14.7%)	<0.001*
*Length of hospital stay*	n = 25	n = 255	
	54.2 ( ± 33.3)	20.3 ( ± 12.7)	<0.001*
*Patients with unknown length of hospital stay were excluded (n* = *285)*.			

Values represent mean (SD; standard deviation) or numbers (%). AIDS, acquired immune deficiency syndrome; COPD, chronic obstructive pulmonary disease; CVA, cerebrovascular accident; CVD, cardiovascular disease (myocardial infarction before intensive-care admission and chronic cardiovascular insufficiency); DM, diabetes mellitus; MIE, minimal invasive esophagectomy; 30D, 30 day mortality; 3 M, 3 month mortality; 1Y, 1 year mortality; APACHE, Acute Physiology and Chronic Health Evaluation; ICU, intensive-care unit. Immunological insufficiencies included long term immunosuppressive therapy; corticosteroid use (both short term high and long term low dosages, e.g. > 5 days 1 mg/kg prednisolone or more than 20 days > 0.1 mg/kg); active chemo- or radiotherapy in the past year; chemo- or radiotherapy for Hodgkin’s or non-Hodgkin’s lymphoma at any time prior to ICU admission; humoral or cellular deficiencies. No malignancies include achalasia, extra lobar pulmonary sequestration and the Boerhaave syndrome. Open esophagectomy included n = 2 patients with a partial resection of the esophagus due to a leiomyoma.

^*^P values < 0.05 were considered significant.

An overview of antifungal treatment is presented in Supplementary Fig. S1. Antifungal treatment did not have a significant effect on mortality (30 day mortality p = 0.305; 3 month mortality p = 0.246; 1 year mortality p = 0.174). Additional surgical intervention was documented for 43.9% (n = 18) of the patients with yeast infections. Almost all patients with a yeast infection did have a drain (n = 39). 30 day mortality was independent of surgery in patients with a drain (p = 1.000). However, these numbers are too small to draw any further conclusions. For a more extensive overview of mortality data, we refer to Supplementary Table S1, where the mortality in the yeast and no yeast infection groups is shown as recorded before 2006, between 2006 and 2014, and after 2014. These specific time periods were chosen because of the introduction of neoadjuvant chemoradiotherapy in 2006 and MIE in 2015. Supplementary Table S2 shows an overview of the pTNM (Tumor Node Metastases; pathology) and ypTNM (pathology after neoadjuvant chemoradiotherapy) classification. We found no relation to more yeast infections with increasing severity of pathological disease.

### Characteristics of yeast infections and drain cultures

**Figure 2:**
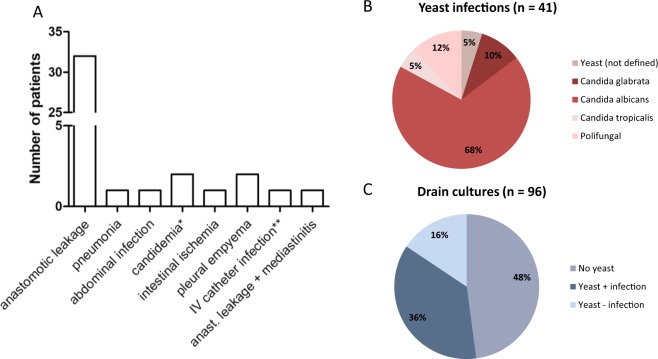
Possible causes and identification of yeast infections. The underlying cause of a yeast infection (**A**), causative species (**B**) and pleural drain cultures with and without yeast (**C**) are shown. In figure C, 3 patients with a yeast infection had a negative pleural drain culture but a positive culture from an alternate site and 3 patients who were defined as infection did not have a pleural drain culture sent for analysis and diagnosis was again made from an alternate site. *Candidemia was only detected in 2 patients. The source of both candidemia cases were unknown. **IV catheter infection without positive blood cultures.

A yeast infection was identified in 41 patients after esophagectomy surgery. Early and late first positive yeast cultures are detailed in the Supplementary Fig. S2. Anastomotic leakage was observed to be the main cause of yeast infection (n = 32; Fig. 2A). Only 2 patients developed candidemia from an unknown source (Fig. 2A). *Candida albicans* was the causative species in the majority of these cases (Fig. 2B). Drain fluid was cultured from 96 patients, mainly because of suspected anastomotic leakage. An overview of the drain cultures in comparison with mortality, survival and readmission to the ICU is presented in Table 2 and Fig. 2C. Table 2 details numbers of cultures with or without yeast growth but not necessarily a treated yeast infection.

**Table 2:** Microbiological analysis of drain cultures.

Variable	(poly)microbial + yeast	(poly)microbial - yeast	P value
*Mortality*	n = 50	n = 46	
30D	5 (10.0%)	2 (4.3%)	0.438
3 M	10 (20.0%)	3 (6.5%)	0.054
1Y	24 (48.0%)	10 (21.7%)	0.070
*Survival* > *1Y*	26 (52.0%)	36 (78.3%)	0.070
*Readmission to ICU*			0.039*
yes	29 (58.0%)	29 (63.0%)	
no	21 (42.0%)	17 (37.0%)	

Values represent numbers (%). 30D, 30 day mortality; 3 M, 3 month mortality; 1Y, 1 year mortality; survival > 1Y, > 1 year survival. Drain cultures with 2 different yeast species without bacterial growth (n = 2) and one yeast species without bacterial growth (n = 6) were included in the (poly)microbial + yeast group. Drain cultures containing one bacterial species (n = 25) were included in the (poly)microbial - yeast group. *P values < 0.05 were considered significant.

### Survival analysis

**Figure 3:**
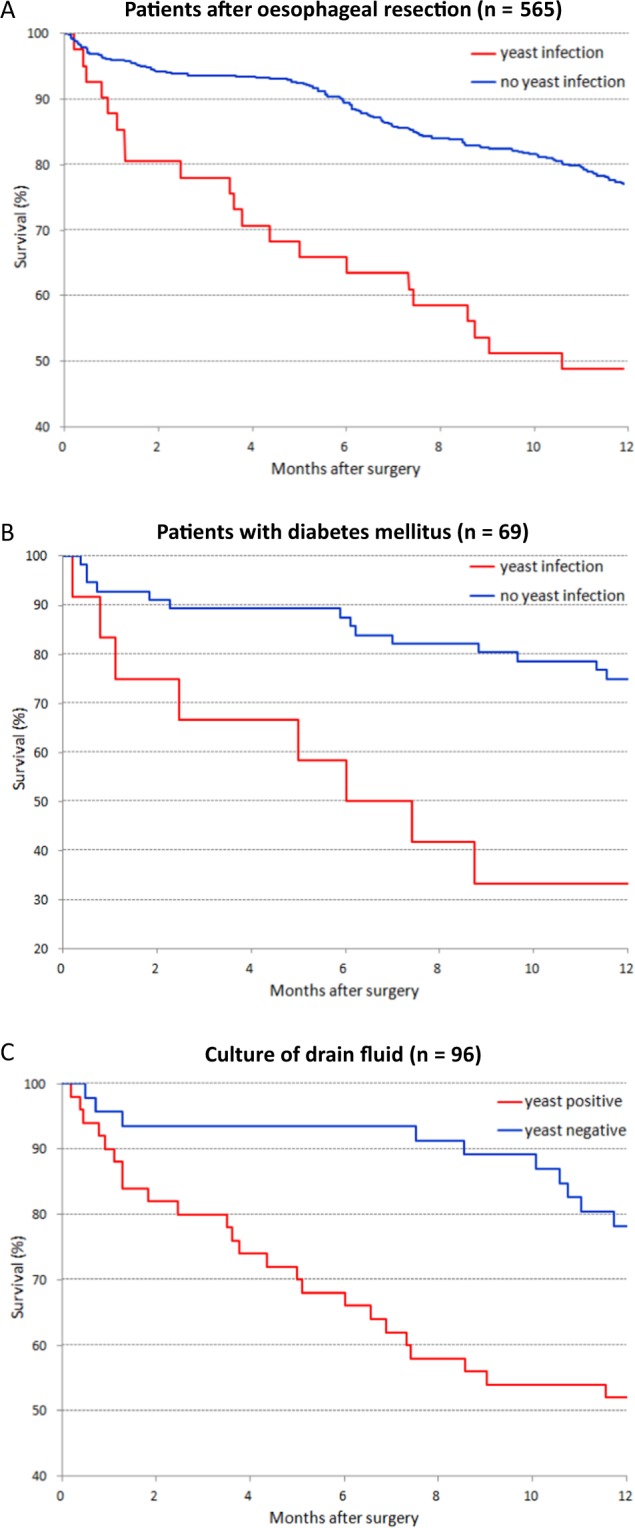
Survival analysis of patients with or without yeast infection. Survival curves of (**A**) patients with yeast infection vs. non-yeast infection, (**B**) yeast infection vs. non-yeast infection in patients with diabetes mellitus, (**C**) patients with (poly)microbial drain culture + yeast vs. (poly)microbial drain cultures - yeast.

The survival of patients with a diagnosed yeast infection was lower compared to patients without a yeast infection (p < 0.001; Fig. 3A), and patients with DM presented a statistically significant increase in overall yeast infection (Table 1). Therefore, a survival analysis was performed to evaluate this patient group (Fig. 3B). Indeed, a higher one-year mortality for DM patients with a yeast infection compared to patients without yeast infection (p = 0.003) was demonstrated. To analyze whether a positive yeast culture in drain fluid from patients with a suspected cervical anastomosis leakage is reflected in the respective patients’ survival, we compared the outcomes of patients with positive or negative yeast culture from polymicrobial drain fluids. Figure 3C shows a substantial reduction in survival of patients whose drain fluid contained a polymicrobial contamination that tested positive upon yeast culture (p = 0.005). Although not all patients with yeast-positive drain cultures were defined as having a yeast infection, the presence of yeast in drain fluid is indicative for an effect on survival.

## Discussion

In this study, we determined the incidence of yeast infections in esophageal resection patients between 1991 and 2017 at the UMCG. 7.3% of patients who underwent esophagectomy developed a yeast infection, the most prevalent organism being *C. albicans*. The most frequent underlying cause was identified as anastomotic leakage. Patients with DM had a statistically significant risk over other detailed comorbidities for developing a yeast infection. Furthermore, there was a significant increase in mortality rates between those in the defined yeast infection versus the non-yeast infection group.

Invasive yeast infections pose a clinical challenge as they have a significant impact on patient outcome, resulting in up to 50% increased mortality^18,19^. Recent studies have shown point prevalence rates of candidemia at 6.9 per 1000 ICU patients with 7.5% of patients requiring systemic antifungal therapy^20^. These figures are from general ICU populations and although comparisons are made with caution, we note similarity to the antifungal therapy required to treat patients included in our study (7.3%). However, the observed candidemia rate was lower compared to the literature (4.9%; n = 2).

Previous studies have shown a correlation between invasive fungal infections and high APACHE II scores^21,22^. However, others have failed to corroborate this link^23^. In our present study, higher APACHE II scores were significantly (p = 0.028) correlated to the development of yeast infection.

Notably, DM was identified as the only chronic disease that showed a statistically significant association to the development of yeast infections. There has long been a multifactorial correlation between DM and both general and surgery-specific infections^24,25^. DM is also a preoperative predictor of mortality in esophagectomy patients with increased morbidity and mortality^5,26^. A systematic review by Li and colleagues demonstrated a link between anastomotic leakage and DM in esophagectomy patients following a similar conclusion in general abdominal surgery patients^27,28^. We identified anastomotic leakage as the most prevalent underlying etiology of complications (Fig. 2), which is consistent with the results of Raymond *et al*. who illustrated 12.0% anastomotic leakage and 12.2% pneumonia in 4321 esophagectomy patients^2^. However, the results of our current study provide no evidence for the same incidence of documented pneumonia. Given the link between cervical anastomotic leakage and pulmonary and polymicrobial infections, the relationship between DM and the development of yeast infection is likely based on a multifaceted interaction. Polymicrobial positive culture from pleural effusion can be logically explained, in this patient category, by cervical anastomotic leakage. Accordingly, the presence of gastro-intestinal microbial flora on pleural fluid culture is usually part of the diagnostic tree. Further, it should be noted that diagnosis of anastomotic leakage is often difficult as current radiological investigations are non-specific. It is therefore conceivable that the prevalence of yeast infection can be attributed to an increased incidence of anastomotic leakage in DM patients.

Patients with a yeast infection showed a 2.5-fold increase in mean length of hospital stay compared to the overall group, which can be explained by the increased therapy and critical care support these patients are known to require. According to the data collected between 2006 and 2014, 8.4% of patients were diagnosed with yeast infections. This is in contrast with data collected after 2014 when only 4.5% of patients developed a yeast infection. We assume that after the introduction of MIE, less yeast infections developed post-esophagectomy. However, the numbers are small and to draw firmer conclusions larger sample sizes would be needed.

There are a few limitations to this study. Firstly, the distinction of yeast infection from colonization is challenging and, accordingly, there is no universally adopted definition^11,29^. Therefore, we have reduced potential diagnostic ambiguity by incorporating current clinical practice and guidelines^30^. It is important to emphasize that the colonized patients were those who had shown a positive yeast culture on routine sampling. However, one should bear in mind that yeast is a component of the human mucosal microbiota despite not always being cultured. It is also necessary to mention the sporadic use of SDD in some patients in our study, which involved the administration of an oral, non-absorbable paste with enteral antibiotics including amphotericin B, tobramycin and colistin, as well as intravenous administration of cefotaxime. This was not a global policy adopted by all of the ICUs within the UMCG and, therefore, the numbers are believed to be small. From October 2005 to April 2006 the SDD-regimen was infrequently used as part of a clinical trial^12^. Additionally, from the summer of 2013, some of this patient cohort may have received SDD. This was introduced as a general infection prevention measure in the ICU following the aforementioned trial, but not used in all esophagectomy patients. Unfortunately, exact numbers are not known since this information relies on subjective documentation by the medical team.

We appreciate the applied definition of yeast infection may lead to potential bias. Our clinical opinion is that patients who commenced therapy were more acutely unwell, which complicates interpretation of our retrospective survival analysis. However, we additionally evaluated the drain fluid with (poly)microbial contamination, presumably derived from patients with anastomotic leakage. These patients are not routinely subject to antifungal therapy. Patients with yeast in their pleural drain fluid showed a diminished survival and therefore, it seems that even the presence of yeast is indicative for survival outcome. However, we recognize this could be the result of increased antimicrobial administration in critically ill patients, resulting in yeast overgrowth. Further, yeast colonization was detected by sputum or throat cultures in the pre- or early post-operative period. From the numbers shown in Table 1, no significant effect of colonization on yeast infection can be inferred. However, the diagnostic microbiology laboratory did not consistently report all yeast in sputum cultures during this period and patients were not routinely screened for yeast colonization. Consequently, not all cases of yeast-colonization will have been detected.

Other limitations to this study are mainly related to the retrospective nature of the work and thus, inherent susceptibility to confounding and bias. Our statistical analysis was designed to take these factors into consideration. Given the electronic patient notes are reliant on user input, we were unable to have complete datasets for all patients. In particular, we were unable to obtain information on pre-operative functional assessment, SDD prophylaxis, smoking status, definitive steroid or H2 histamine receptor blocker administration, total parenteral nutrition and alcohol intake for all the subjects. Moreover, the use of antimicrobial therapy in addition to SDD was sourced from ICU discharge letters, overall admission discharge letters and the reports from the consulted clinical microbiologist. As the clinical microbiologist was contacted for all patients with yeast infections, the antimicrobial therapy is believed to be accurate for this group.

Lastly, SDD therapy is mainly applied in the Netherlands, because it is supported by several prospective, multi-center randomized controlled trials^10,12^. However, there has been no prospective investigation on whether antifungal prophylaxis, especially pre-operatively, should be used in esophagectomy patients. Although the present results should be interpreted with caution, our findings encourage further prospective investigations on the possible benefits of antifungal cover in the SDD regimen of esophagectomy patients.

## Methods

### Study design and population

At the UMCG we carried out a retrospective analysis of all esophagectomy patients between January 1991 and July 2017. In total, there were 616 surgical procedures. We analyzed 565 patients. Patients with incomplete data sets (i.e. laboratory outcomes (n = 49)) and unknown comorbidities (n = 2) were excluded (Fig. 1). We distinguished patients who developed a yeast infection after surgery and those who did not. During the study period, patients admitted to the UMCG complied with hospital guidelines in an opt-out research consent procedure. Consequently, individual written consent was not required for the inclusion of these patients to our retrospective study. Unless stated otherwise in the medical file, consent was given through this opt-out practice. Permission for this study, including the opt-out patient consent protocol, was obtained from the Medical Ethical Review Board Committee (Medisch Ethische Toetsingscommissie METc) UMCG, and all collected data was treated pseudo-anonymously and in adherence with the Declaration of Helsinki.

### Definitions

Yeast colonization was defined as a positive sputum and/or throat culture, or yeast detected in a Gram staining of sputum 1 day preoperative until 3 days postoperative. A yeast infection was defined as a positive yeast culture in primarily sterile materials (including pus, drain, pleural and wound fluid) with subsequent treatment of the patient with antifungal therapy during hospitalization. Additionally, a yeast positive blood culture or a positive culture of a sterile site after autopsy was regarded as a yeast infection.

### Data collection

Data was obtained via the electronic health record systems of the UMCG (PoliPlus until November 2017 and Electronic Patient Dossier (EPD) from December 2017 onward) and the laboratory information systems of the Department of Medical Microbiology (General Laboratory Information Management System; GLIMS and ‘Bacteriologisch Ziekenhuisinformatiesysteem’; BACZIS). Several demographic characteristics, microbiological results and patient comorbidities were identified. Moreover, surgery-specific information, pathological diagnosis and ICU admission criteria and subsequent mortality figures were also collected. The National Intensive Care Evaluation database (NICE) and the national pathology database (PALGA; ‘Pathologisch-Anatomisch Landelijk Geautomatiseerd Archief’) were additional information sources consulted.

### Statistical analysis

Unpaired t-tests, Mann-Whitney U tests and Chi square were performed using IBM SPSS Statistics 23. The Fisher’s exact test was used when >20% cells had an expected count less than 5. P-values of 0.05 or less were considered significant. Aforementioned tests were used to compare the data of the two groups (yeast infection vs. no yeast infection and (poly)microbial + yeast vs. (poly)microbial - yeast). To test for trend we used the Mantel-Haenszel test. Specifically, to assess a possible higher yeast infection risk with increasing severity of pathological disease, a binary logistic regression analysis was performed with three TNM (pathology) classes, Tis-I, II and III-IV as a linear variable in the equation and yeast infection as outcome variable. One-year survival differences were tested for significance with Cox regression analysis.

## Supplementary information

Supplementary data.

## References

[1] Biere SS (2012). Minimally invasive versus open oesophagectomy for patients with oesophageal cancer: a multicentre, open-label, randomised controlled trial. Lancet.

[2] Raymond DP (2017). Predictors Of Major Morbidity Or Mortality After Resection For Esophageal Cancer: A Society Of Thoracic Surgeons General Thoracic Surgery Database Risk Adjustment Model. HHS Public. Access..

[3] Sjoquist KM (2011). Survival after neoadjuvant chemotherapy or chemoradiotherapy for resectable oesophageal carcinoma: an updated meta-analysis. Lancet Oncol..

[4] Atkins BZ (2004). Reducing Hospital Morbidity and Mortality Following Esophagectomy. Ann. Thorac. Surg..

[5] Wright CD, Kucharczuk JC, O’Brien SM, Grab JD, Allen MS (2009). Predictors of major morbidity and mortality after esophagectomy for esophageal cancer: A Society of Thoracic Surgeons General Thoracic Surgery Database risk adjustment model. J. Thorac. Cardiovasc. Surg..

[6] Wang S-L (2006). Investigation of Clinical and Dosimetric Factors Associated with Postoperative Pulmonary Complications in Esophageal Cancer Patients Treated with Concurrent Chemoradiotherapy Followed by Surgery. Int. J. Radiat. Oncol. Biol. Phys..

[7] Tandon S (2001). Peri-operative risk factors for acute lung injury after elective oesophagectomy. Br. J. Anaesth..

[8] Yapar N (2014). Epidemiology and risk factors for invasive candidiasis. Ther. Clin. Risk Manag..

[9] Ong DSY, Klein Klouwenberg PMC, Spitoni C, Bonten MJM, Cremer OL (2013). Nebulised amphotericin B to eradicate Candida colonisation from the respiratory tract in critically ill patients receiving selective digestive decontamination: a cohort study. Crit. Care.

[10] Hurley JC (2016). Impact of selective digestive decontamination on respiratory tract Candida among patients with suspected ventilator-associated pneumonia. A meta-analysis. Eur. J. Clin. Microbiol. Infect. Dis..

[11] van Till JWO (2007). Single-drug Ther. selective decontamination digestive tract. antifungal prophylaxis critically ill. patients: a Syst. Rev..

[12] de Smet AMGA (2009). Decontamination of the Digestive Tract and Oropharynx in ICU Patients. N. Engl. J. Med..

[13] Bergmans DCJJ (2001). Prevention of Ventilator-associated Pneumonia by Oral Decontamination: a prospective, randomized, double-blind, placbo-controlled study. Am. J. Respir. Crit. Care Med..

[14] Melsen WG, de Smet AMGA, Kluytmans JAJW, Bonten MJM (2012). Selective decontamination of the oral and digestive tract in surgical versus non-surgical patients in intensive care in a cluster-randomized trial. Br. J. Surg..

[15] Plantinga NL (2018). Selective digestive and oropharyngeal decontamination in medical and surgical ICU patients: individual patient data meta-analysis*. Clin. Microbiol. Infect..

[16] Roos D (2011). Randomized clinical trial of perioperative selective decontamination of the digestive tract versus placebo in elective gastrointestinal surgery. Br. J. Surg..

[17] de Smet AMGA, Bonten MJM, Kluytmans JAJW (2012). For whom should we use selective decontamination of the digestive tract?. Curr. Opin. Infect. Dis..

[18] Gudlaugsson O (2003). Attributable Mortality of Nosocomial Candidemia, Revisited. Clin. Infect. Dis..

[19] Arendrup MC (2011). Diagnostic issues, clinical characteristics, and outcomes for patients with fungemia. J. Clin. Microbiol..

[20] Azoulay E (2012). Systemic antifungal therapy in critically ill patients without invasive fungal infection*. Crit. Care Med..

[21] Jordà-Marcos R (2007). Risk factors for candidaemia in critically ill patients: A prospective surveillance study. Mycoses.

[22] Agvald-Öhman C, Klingspor L, Hjelmqvist H, Edlund C (2008). Invasive candidiasis in long-term patients at a multidisciplinary intensive care unit: Candida colonization index, risk factors, treatment and outcome. Scand. J. Infect. Dis..

[23] Borzotta AP, Beardsley K (1999). Candida Infections in Critically Ill Trauma Patients: A Retrospective Case-Control Study. Arch. Surg..

[24] Shah BR, Hux JE (2003). Quantifying the risk of infectious diseases for people with diabetes. Diabetes Care.

[25] Martin ET (2016). Diabetes and risk of surgical site infection: A systematic review and meta-analysis. Infect. Control. Hosp. Epidemiol..

[26] Bailey S. H. *et al*. Outcomes after esophagectomy: a ten-year prospective cohort. *Ann. Thorac. Surg.* **75**, 217–22, discussion 222 (2003). 10.1016/s0003-4975(02)04368-0 12537219

[27] Li SJ (2017). Diabetes mellitus and risk of anastomotic leakage after esophagectomy: a systematic review and meta-analysis. Dis. Esophagus.

[28] Lin X (2015). Diabetes and risk of anastomotic leakage after gastrointestinal surgery. J. Surg. Res..

[29] van der Geest PJ, Dieters EI, Rijnders B, Groeneveld JAB (2014). Safety and efficacy of amphotericin-B deoxycholate inhalation in critically ill patients with respiratory Candida spp. colonization: A retrospective analysis. BMC Infect. Dis..

[30] Arendrup MC (2014). ESCMID and ECMM joint clinical guidelines for the diagnosis and management of rare invasive yeast infections. Clin. Microbiol. Infect..

