# Supplementary material for: Yeast Infections after Esophagectomy: A Retrospective Analysis
Source: Sci Rep. 2020 Mar 9;10:4343. doi: 10.1038/s41598-020-61113-z (PMC7062806; doi:10.1038/s41598-020-61113-z)
Supplement: Supplementary file 1 — Supplementary data. [file 41598_2020_61113_MOESM1_ESM.docx]

**Yeast Infections after Esophagectomy: A Retrospective Analysis**

Marjolein Heuker^1#^, Usma Koser^2#^, Alewijn Ott^3^, Arend Karrenbeld^4^, Jan Maarten van Dijl^1^, Gooitzen M. van Dam^2,5^, Anne Marie G.A. de Smet^2^, Marleen van Oosten^1*^

^#^ Both authors contributed equally to this work.

^1^Department of Medical Microbiology, University of Groningen, University Medical Center Groningen, Hanzeplein 1, PO Box 30001, 9700 RB Groningen, The Netherlands

^2^ Intensive Care Unit, University of Groningen, University Medical Center Groningen, Hanzeplein 1, PO Box 30001, 9700 RB Groningen, The Netherlands

^3^ Department of Medical Microbiology, Certe, PO Box 909, 9700 AX Groningen, The Netherlands

^4^ Department of Pathology, University of Groningen, University Medical Center Groningen, Hanzeplein 1, PO Box 30001, 9700 RB Groningen, The Netherlands

^5^ Department of Surgery, Division of Surgical Oncology, University of Groningen, University Medical Center Groningen, Hanzeplein 1, PO Box 30001, 9700 RB Groningen, The Netherlands

**^*^ Corresponding author:** Marleen van Oosten, Department of Medical Microbiology, University Medical Center Groningen, Hanzeplein 1, PO BOX 30001, 9700 RB Groningen, The Netherlands. Telephone: +31 50 361 3480. Fax: +31 50 361 9150. Email: m.van.oosten@umcg.nl

**Running Title:** Yeast Infections after Esophagectomy

**Supplementary data**

**Table S1. Mortality**

| mortality | yeast infection | no yeast infection | P value |
| --- | --- | --- | --- |
| Total | n = 41 | n = 522 |  |
| 30D | 5 (12.2%) | 21 (4.0%) | 0.039* |
| 3M | 9 (22.0%) | 33 (6.3%) | 0.002* |
| 1Y | 21 (51.2%) | 118 (22.6%) | 0.000* |
|  | | | |
| < 2006 | n = 9 | n = 120 |  |
| 30D | 1 (11.1%) | 7 (5.8%) | 0.449 |
| 3M | 3 (33.3%) | 10 (8.3%) | 0.047* |
| 1Y | 7 (77.8%) | 22 (18.3%) | 0.000* |
|  |  |  |  |
| 2006-2014 | n = 27 | n = 296 |  |
| 30D | 4 (14.8%) | 10 (3.4%) | 0.022* |
| 3M | 6 (22.2%) | 17 (5.7%) | 0.007* |
| 1Y | 13 (48.1%) | 67 (22.6%) | 0.003* |
|  |  |  |  |
| > 2014 | n = 5 | n = 106 |  |
| 30D | 0 (0.0%) | 5 (4.7%) | 1.000 |
| 3M | 0 (0.0%) | 7 (6.6%) | 1.000 |
| 1Y | 1 (20.0%) | 30 (28.3%) | 1.000 |
| *Patients with unknown date of death were excluded (n = 2).* | | | |

Values represent numbers (%). 30D, 30 day mortality; 3M, 3 month mortality; 1Y, 1 year mortality. ^*^P values <0.05 were considered significant.

**Table S2. Pathology classification**

| variable | yeast infection | no yeast infection |
| --- | --- | --- |
| *pTNM classification* |  |  |
| Adenocarcinoma | n = 17 | n = 212 |
| Tis | 0 (0.0%) | 10 (4.7%) |
| IA | 0 (0.0%) | 9 (4.2%) |
| IB | 0 (0.0%) | 12 (5.7%) |
| IC | 2 (11.8%) | 24 (11.3%) |
| IIA | 1 (5.9%) | 11 (5.2%) |
| IIB | 3 (17.6%) | 28 (13.2%) |
| IIIA | 2 (11.8%) | 18 (8.5%) |
| IIIB | 7 (41.2%) | 86 (40.6%) |
| IVA | 2 (11.8%) | 14 (6.6%) |
|  |  |  |
| Squamous cell carcinoma | n = 7 | n = 47 |
| IA | 0 (0.0%) | 1 (2.1%) |
| IB | 0 (0.0%) | 8 (17.0%) |
| IIA | 1 (14.3%) | 4 (8.5%) |
| IIB | 1 (14.3%) | 10 (21.3%) |
| IIIA | 0 (0.0%) | 2 (4.3%) |
| IIIB | 5 (71.4%) | 18 (38.3%) |
| IVA | 0 (0.0%) | 3 (6.4%) |
| IVB | 0 (0.0%) | 1 (2.1%) |
|  |  |  |
| *ypTNM classification* | n = 14 | n = 239 |
| I | 3 (21.4%) | 102 (42.7%) |
| II | 3 (21.4%) | 53 (22.2%) |
| IIIA | 2 (14.3%) | 23 (9.6%) |
| IIIB | 6 (42.9%) | 51 (21.3%) |
| IVA | 0 (0.0%) | 8 (3.3%) |
| IVB | 0 (0.0%) | 2 (0.8%) |
| *Patients with T0 without neoadjuvant chemoradiotherapy were excluded (n = 8)* | | |

Values represent numbers (%). pTNM, pathology TNM classification; ypTNM, pathology TNM classification after neoadjuvant chemoradiotherapy. We found no relation of more yeast infections with increasing severity of pathological disease (p = 0.35).

**Supplementary Figure 1.** **Antifungal treatment**

**
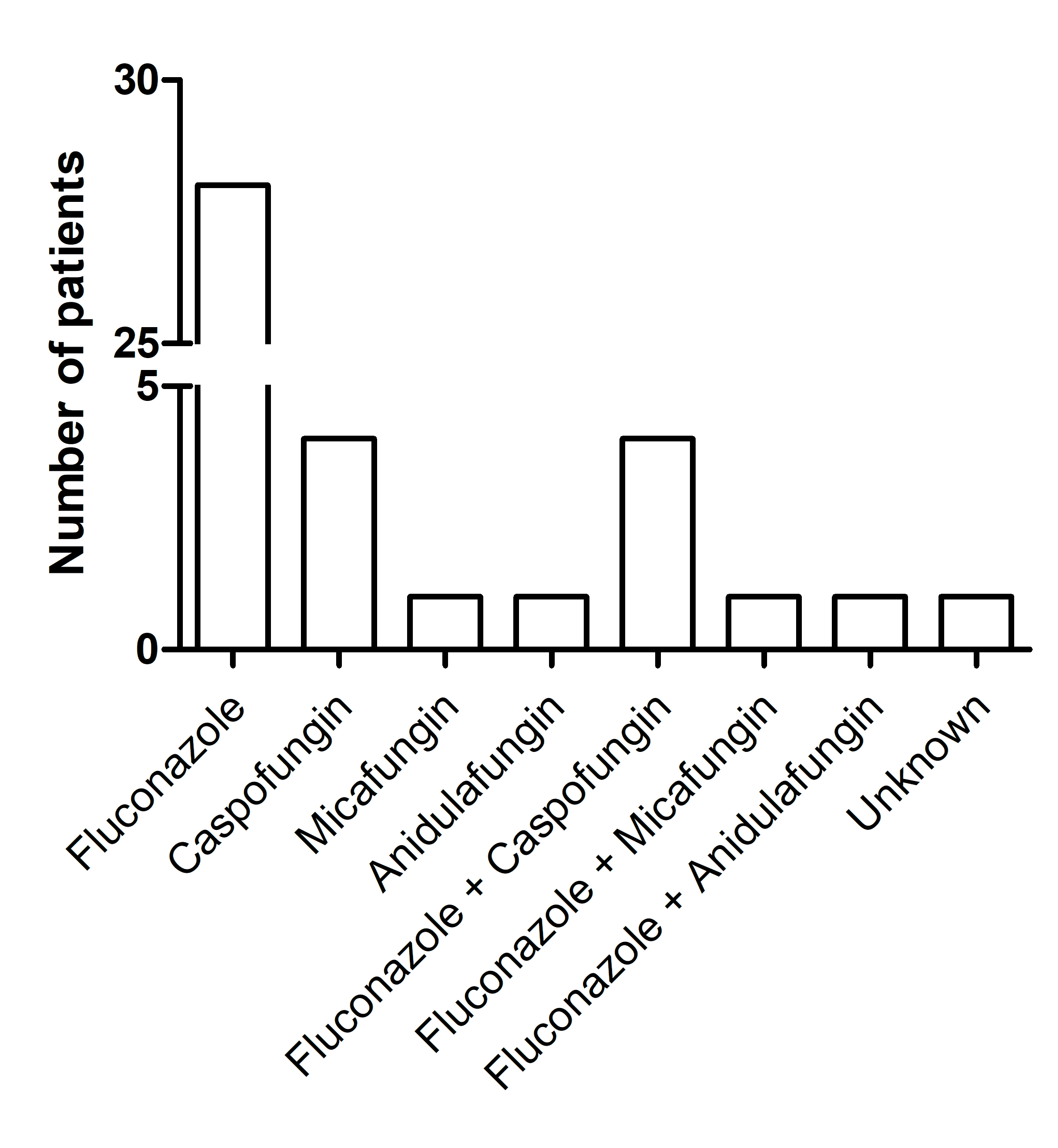
**

**Supplementary Figure 2. Day in admission of first yeast-positive culture**

**
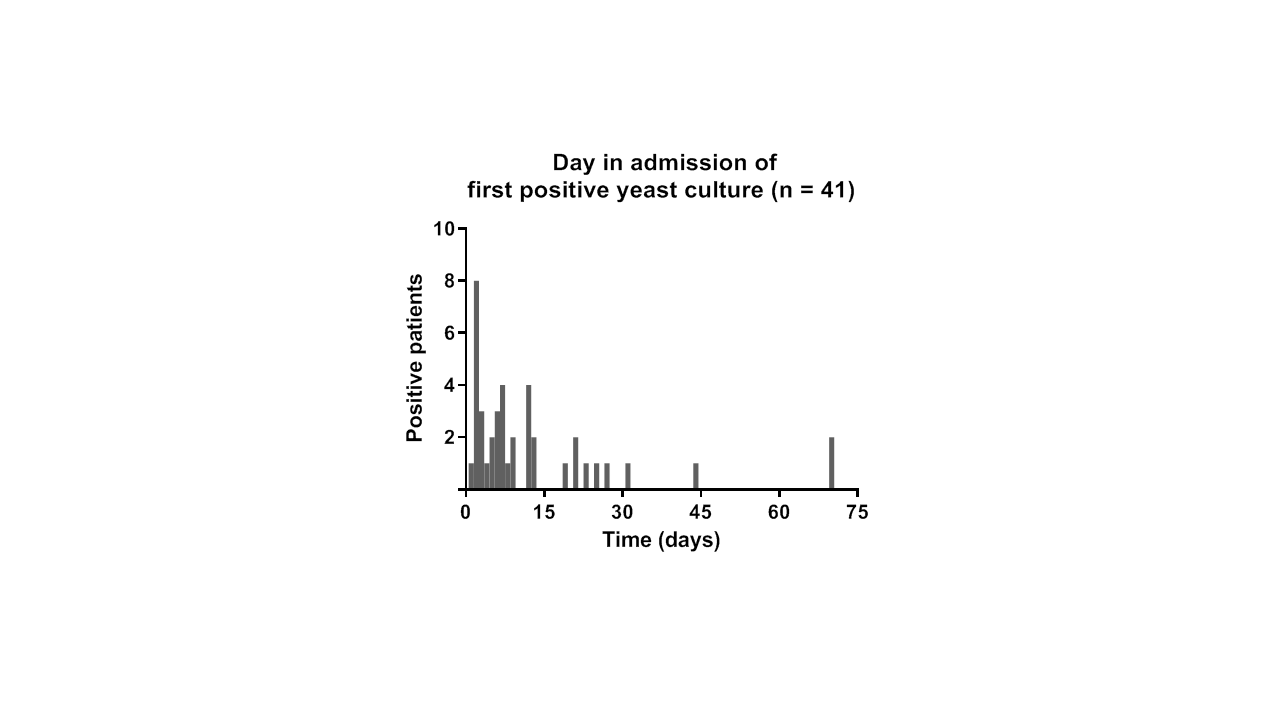
**

**Figure legends**

**Supplementary Figure 1.** **Antifungal treatment.** Type of antifungal therapy in patients with a yeast infection (n = 41).

**Supplementary Figure 2. Day in admission of first yeast-positive culture.** The diagram scores the number of days after admission when a first positive yeast culture was obtained from patients with a yeast infection (n = 41).
